# Insulin autoimmune hypoglycemia unmasking monoclonal gammopathy in type 2 diabetes

**DOI:** 10.1210/jcemcr/luag190

**Published:** 2026-07-13

**Authors:** Sai Prasad, Anukriti Sharma, Vidhi Parmar, Tanisha Jindal, Darpan Kothia, Deep Bavaria

**Affiliations:** Department of Internal Medicine, S. Nijalingappa Medical College, Navanagar, Bagalkote, Karnataka 587102, India; Department of Internal Medicine, Jaipur National University Institute of Medical Sciences and Research Centre, Jaipur, Rajasthan 302017, India; Department of Endocrinology, Mayo Clinic Rochester, Rochester, MN 55905, USA; Department of Internal Medicine, Topiwala National Medical College, Mumbai, Maharashtra 400008, India; Department of Internal Medicine, Ascension Sacred Heart Pensacola, Florida State University, Pensacola, FL 32504, USA; Department of Internal Medicine, MP shah Government Medical College, Jamnagar, Gujarat 361006, India

**Keywords:** insulin autoimmune syndrome, Hirata disease, monoclonal gammopathy, MGUS, hypoglycemia

## Abstract

Insulin autoimmune syndrome (IAS), also known as Hirata disease, is a rare cause of spontaneous hypoglycemia due to circulating insulin autoantibodies, most commonly described in East Asian individuals and those exposed to sulfhydryl-containing drugs. We describe a 58-year-old South Asian man with type 2 diabetes mellitus who presented with recurrent fasting and postprandial hypoglycemia despite cessation of all glucose-lowering medications. A spontaneous episode confirmed Whipple triad: plasma glucose 38 mg/dL (SI: 2.1 mmol/L; reference range, 70-99 mg/dL [SI: 3.9-5.5 mmol/L]), markedly elevated serum insulin at 156 µIU/mL (SI: 1083 pmol/L; reference range, 2.6-24.9 µIU/mL [SI: 18-173 pmol/L]), and C-peptide of 5.8 ng/mL (SI: 1.9 nmol/L; reference range, 0.5-2.0 ng/mL [SI: 0.17-0.66 nmol/L]), with a negative sulfonylurea screen and markedly elevated insulin autoantibody titers. Polyethylene glycol precipitation confirmed antibody-bound insulin sequestration. Workup identified IgG-kappa monoclonal gammopathy of undetermined significance as the probable precipitant. Dietary modification, oral glucocorticoids, and acarbose achieved near-complete resolution of hypoglycemia with declining autoantibody titers over 4 weeks. This case highlights the importance of considering IAS in unexplained hyperinsulinemic hypoglycemia and the need to screen for plasma cell dyscrasias in atypical presentations.

## Introduction

In most instances, hypoglycemia in patients with type 2 diabetes is attributed to glucose-lowering therapy. However, when episodes persist after drug withdrawal, clinicians are compelled to broaden the differential diagnosis considerably. Endogenous hyperinsulinism due to insulinoma, autoimmune hypoglycemia, adrenal insufficiency, and paraneoplastic processes warrants consideration. Among these, insulin autoimmune syndrome (IAS) occupies a particularly deceptive niche, as its biochemical parameters mimic those of an insulin-secreting tumor, yet the mechanism is entirely antibody-mediated.

Insulin autoimmune syndrome was first described by Hirata et al in 1970 in Japan and has since been recognized across diverse ethnic groups, although it remains disproportionately prevalent among individuals of East Asian descent [1]. The fundamental pathophysiology involves high-affinity immunoglobulins that bind to endogenous insulin, creating a large reservoir of antibody-bound hormones. Insulin is sequestered postprandially when secretion is brisk and is released unpredictably in the fasting state, provoking hypoglycemia at times unrelated to meals [2].

Drug exposure, particularly to sulfhydryl-containing compounds such as methimazole, captopril, and α-lipoic acid, accounts for most cases outside Japan [3]. An increasingly recognized but still underreported trigger is monoclonal immunoglobulin production, as occurs in monoclonal gammopathy of undetermined significance (MGUS) and smoldering or overt multiple myeloma. The monoclonal protein, by virtue of its immunoglobulin structure, may possess insulin-binding properties that can drive IAS [4]. This association is clinically important because the management of the underlying plasma cell dyscrasia may ultimately determine the trajectory of hypoglycemia.

We present a case of IAS in a man with preexisting type 2 diabetes, in whom a comprehensive evaluation ultimately identified MGUS as the probable precipitant.

## Case presentation

A 58-year-old man of South Asian ethnicity with a 10-year history of type 2 diabetes mellitus, previously managed with metformin 1000 mg twice daily and glipizide 5 mg daily, was referred for evaluation of recurrent hypoglycemia of 3 weeks' duration. The episodes occurred in both fasting and postprandial states, manifesting as diaphoresis, tremulousness, and episodic confusion. Home capillary glucose readings during symptomatic periods ranged from 32 to 54 mg/dL (SI: 1.8-3.0 mmol/L).

There was no history of exogenous insulin use, recent changes in oral hypoglycemic dosing, excessive alcohol consumption, significant weight loss, or concurrent illnesses. Crucially, all glucose-lowering agents were discontinued 2 weeks prior to presentation by his general practitioner; however, the hypoglycemic episodes persisted unabated. He had no history of autoimmune disease, thyroid dysfunction, adrenal insufficiency, or prior exposure to methimazole, captopril, or α-lipoic acid. The family history was noncontributory.

On examination, the patient was alert but diaphoretic during the episode. The vital signs were stable. There was no evidence of clinical lymphadenopathy, hepatosplenomegaly, or adrenal insufficiency. The blood glucose level at the time of examination was 38 mg/dL (SI: 2.1 mmol/L).

## Diagnostic assessment

Venous blood drawn during the spontaneous hypoglycemic episode demonstrated plasma glucose of 38 mg/dL (SI: 2.1 mmol/L) with markedly elevated serum insulin at 156 µIU/mL (SI: 1083 pmol/L) and C-peptide of 5.8 ng/mL (SI: 1.9 nmol/L), satisfying Whipple triad (symptomatic hypoglycemia, documented low plasma glucose, and resolution of symptoms with glucose correction). Notably, the patient had experienced postprandial glucose excursions followed by rebound hypoglycemia in the fasting state, a pattern characteristic of IAS, wherein antibody-bound insulin is released unpredictably after postprandial sequestration. Proinsulin levels were elevated, and β-hydroxybutyrate levels were appropriately suppressed, consistent with hyperinsulinism rather than starvation or counterregulatory failure. A comprehensive sulfonylurea and meglitinide screen was negative, excluding secretagogue-mediated insulin release.

The disproportionate elevation of total serum insulin relative to C-peptide raised immediate suspicion of antibody-mediated interference. In physiological insulin secretion, insulin and C-peptide are released in equimolar ratios. Circulating insulin autoantibodies artificially inflate immunoassay-measured insulin levels by forming antigen–antibody complexes captured in conventional assays, whereas C-peptide levels remain comparatively modest, as they are unaffected by antibody binding. Insulin autoantibody titers were subsequently confirmed to be markedly elevated at >30 U/mL (reference range <1 U/mL).

Polyethylene glycol precipitation testing was performed to quantify the fraction of free insulin. Following precipitation of immunoglobulin-bound insulin, free insulin fell to 8 µIU/mL, confirming that the overwhelming majority of measured insulin was antibody-bound. This finding is pathognomonic of IAS and distinguishes it from insulinoma, in which free insulin levels are genuinely elevated.

Given the atypical presentation and absence of drug exposure, a systematic search for an underlying hematological cause was undertaken. Thyroid function, morning cortisol, and adrenocorticotropic hormone stimulation tests were all within normal limits. Autoimmune screening, including for antinuclear antibody and anti-dsDNA, was negative. Serum protein electrophoresis revealed a discrete M-protein spike in the gamma region, immunofixation electrophoresis identified an IgG-kappa monoclonal protein, and free light chain assay showed an elevated kappa to lambda ratio of 8.4 (reference 0.26-1.65). Serum calcium, lactate dehydrogenase, and renal functions were normal. A skeletal survey revealed no lytic lesions. In the absence of criteria for overt multiple myeloma, a diagnosis of MGUS was established according to the International Myeloma Working Group criteria.

Contrast-enhanced computed tomography of the pancreas and subsequent endoscopic ultrasound revealed no discrete pancreatic mass or lesion suggestive of insulinoma, further supporting an autoimmune etiology.

## Treatment

Initial management prioritizes the prevention of further hypoglycemic episodes through structured dietary interventions. The patient was advised to consume small, frequent meals composed predominantly of low-glycemic index complex carbohydrates and to avoid prolonged fasting. Rapidly absorbable glucose in the form of dextrose gel was administered for breakthrough symptomatic episodes.

Given the persistence and severity of hypoglycemia, pharmacological immunosuppression was initiated with oral prednisolone 30 mg daily to reduce autoantibody production and improve insulin kinetics. Within 1 week, the frequency of symptomatic episodes declined substantially, and home glucose readings stabilized above 70 mg/dL (SI: 3.9 mmol/L).

Acarbose 50 mg thrice daily with meals was added as adjunctive therapy to blunt postprandial glucose absorption and attenuate the surge in endogenous insulin secretion that fuels antibody loading. All sulfonylurea therapies were permanently discontinued. The hematology team was engaged for a multidisciplinary review, and given the MGUS designation and absence of end-organ damage, a conservative watch-and-wait strategy was adopted. Prednisolone was tapered over 4 weeks with close monitoring of glucose profiles and autoantibody titers.

## Outcome and follow-up

The patient achieved near-complete resolution of hypoglycemic episodes within 2 weeks of initiating combined dietary and pharmacological therapies. Serial capillary glucose monitoring during the tapering phase showed sustained glycemic stability without symptomatic episodes. Repeat biochemical testing at 4 weeks demonstrated a progressive decline in insulin autoantibody titers to 6.2 U/mL, accompanied by the normalization of serum-free insulin levels.

**Figure 1:**
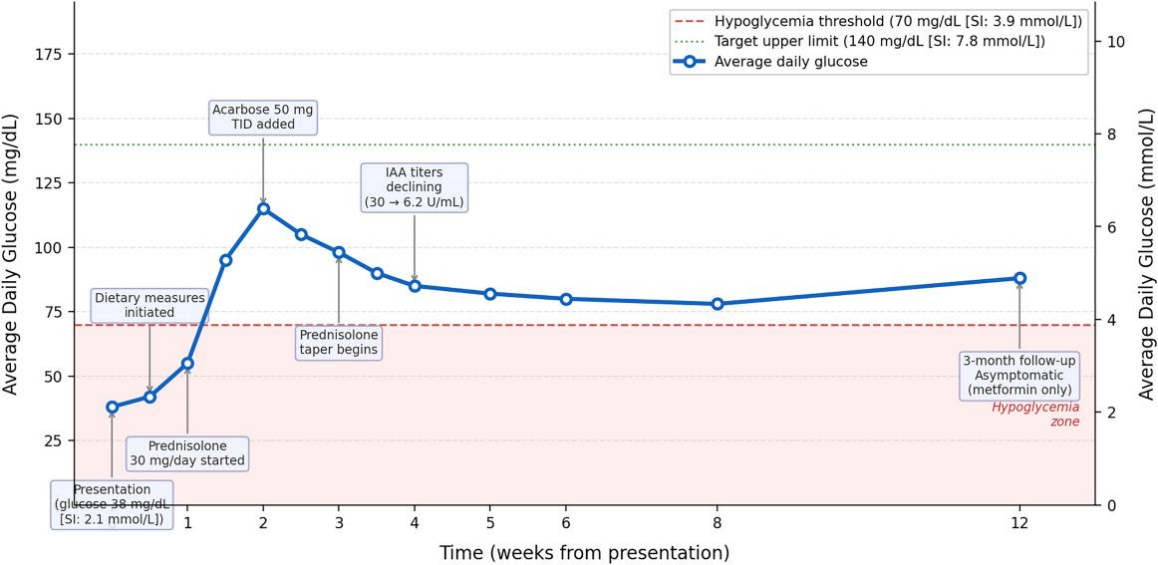
Glucose trend and clinical events over the course of management of insulin autoimmune syndrome. The shaded red zone indicates the hypoglycemia range (<70 mg/dL [SI: <3.9 mmol/L]). Key clinical events are annotated at the corresponding timepoints. IAA, insulin autoantibody; TID, three times daily.

Glycemic management was maintained with metformin monotherapy at 1000 mg twice daily without recurrence of hypoglycemia. At 3-month follow-up, the patient remained asymptomatic. Serum protein electrophoresis showed no significant changes in M-protein concentration. The hematology team confirmed no new end-organ features, and periodic surveillance at 6-month intervals was planned in accordance with MGUS monitoring guidelines (Fig. 1).

## Discussion

This case illustrates a diagnostically challenging scenario in which IAS emerged in a patient with established type 2 diabetes, no prior insulin exposure, and no identifiable drug triggers. The clinical significance lies in several converging issues: the rarity of IAS in non-Japanese populations, the potential for monoclonal immunoglobulins to drive insulin binding, and the tendency for elevated insulin levels in diabetic patients to be attributed reflexively to medication.

Insulin autoimmune syndrome is the third most common cause of spontaneous hypoglycemia in Japan; however, it is far less prevalent in Western populations, where drug exposure most often acts as the precipitant [1, 3]. The cardinal biochemical hallmark is a disproportionately elevated total serum insulin level relative to C-peptide, which reflects antibody-mediated assay interference rather than true pancreatic hypersecretion [5]. Polyethylene glycol precipitation, as performed in this case, remains an indispensable confirmatory step as it provides a functional estimate of free insulin that guides clinical interpretation and directly distinguishes IAS from insulinoma [6].

The association between IAS and hematological disorders involving abnormal immunoglobulin production is well established, albeit underappreciated in everyday practice. Case series have documented IAS in the context of MGUS, smoldering myeloma, Waldenstrom macroglobulinemia, and overt multiple myeloma [4, 7]. The putative mechanism involves a monoclonal immunoglobulin whose complementarity-determining regions possess a structural affinity for the insulin molecule. This is not merely an epiphenomenon; in reported cases, treatment directed at the underlying clonal plasma cell population has led to durable remission of autoimmune hypoglycemia, reinforcing the pathogenic link [7]. The resolution of hypoglycemia in this patient despite no specific treatment directed at MGUS and an unchanged M-protein level warrants comment. The improvement observed likely reflects the immunomodulatory effect of glucocorticoids, which suppress overall immunoglobulin production, potentially including the insulin-binding monoclonal antibody. It is plausible that the quantity or avidity of insulin-binding immunoglobulin was reduced by corticosteroid therapy without necessarily altering the total M-protein concentration detectable on electrophoresis. This dissociation between M-protein stability and clinical remission has been observed in other MGUS-associated IAS cases and does not negate the pathogenic role of the monoclonal protein; rather, it highlights that IAS-directed immunosuppression can be effective even when the underlying clonal process remains stable.

In the present case, the IgG-kappa monoclonal protein was identified only because IAS prompted a systematic search for immune dysregulation. If the diagnosis of IAS had not been made, MGUS would have gone undetected, with attendant implications for future myeloma surveillance. This illustrates a bidirectional diagnostic dividend: recognizing IAS should prompt hematological screening, while a known plasma cell dyscrasia should raise the index of suspicion for autoimmune hypoglycemia in any patient presenting with unexplained low glucose levels. Monoclonal gammopathy of undetermined significance was diagnosed in accordance with the International Myeloma Working Group criteria, which require the absence of myeloma-defining events [8]. The novelty of this case lies in several important dimensions. First, IAS occurring in the context of MGUS in a South Asian individual with type 2 diabetes represents a double diagnostic rarity: IAS itself is uncommon outside East Asian populations, and its association with MGUS in a patient with preexisting diabetes creates a phenotype that is virtually unreported. Second, this case demonstrates that the index diagnosis of IAS can unmask an occult hematological disorder with independent long-term surveillance implications. Third, the demonstration of clinical remission with corticosteroids despite stable M-protein levels contributes to the evolving understanding of the relationship between monoclonal immunoglobulin production and insulin autoantibody pathophysiology. These converging features make this case a meaningful addition to the literature on MGUS-associated IAS.

Glucocorticoids are the most widely used first-line pharmacological intervention, with responses documented in multiple case series [9]. Acarbose exploits the postprandial mechanics of the IAS by reducing intestinal glucose absorption, thereby dampening the insulin secretory stimulus and limiting antibody loading [10]. For refractory cases or those in which the underlying plasma cell dyscrasia is progressive, treatment escalation to rituximab, plasmapheresis, or myeloma-directed chemotherapy has been reported [7].

Finally, this case serves as a reminder that hypoglycemia in patients with type 2 diabetes who are not on insulin should always trigger a structured diagnostic evaluation. Persistence of hypoglycemia after drug withdrawal must be treated as a red flag requiring the full Whipple triad workup, including measurement of insulin, C-peptide, proinsulin, β-hydroxybutyrate, and a sulfonylurea screen, before embarking on an investigation for rarer etiologies, including IAS.

## Learning points

- Insulin autoimmune syndrome should be considered in any patient with unexplained hyperinsulinemic hypoglycemia, particularly when episodes persist after the withdrawal of all glucose-lowering drugs and no exogenous insulin has been administered. The hallmark biochemical finding is a disproportionately elevated total serum insulin level relative to C-peptide, reflecting antibody interference rather than true pancreatic hypersecretion.
- Polyethylene glycol precipitation testing is a critical confirmatory investigation for suspected IAS. By precipitating immunoglobulin-bound insulin, it quantifies the free insulin fraction and directly demonstrates antibody-mediated sequestration, distinguishing IAS from insulinoma and other causes of endogenous hyperinsulinism.
- Monoclonal gammopathy, including MGUS, is an under-recognized secondary cause of IAS. All patients diagnosed with IAS without an obvious drug trigger should undergo serum protein electrophoresis and immunofixation testing. Conversely, patients with a known plasma cell dyscrasia who present with hypoglycemia should be evaluated for IAS.
- First-line management involves dietary modification with frequent small meals, oral glucocorticoids to suppress autoantibody production, and adjunctive acarbose to attenuate postprandial insulin surges. In cases associated with monoclonal gammopathy, a multidisciplinary approach involving hematology is essential, as treatment of the underlying clonal process may be required for durable remission.
- In patients with type 2 diabetes, hypoglycemia persisting after the cessation of all glucose-lowering therapies must not be attributed to residual drug effects. A structured evaluation using Whipple triad and targeted biochemical testing is mandatory to identify rare but treatable causes, such as IAS, insulinoma, or cortisol deficiency.

## Contributors

All authors made substantial individual contributions to authorship. S.P., A.S., and T.J. were involved in the clinical evaluation, diagnosis, and management of the patient. V.P. contributed to the endocrinological assessment and interpretation of biochemical findings. D.K. and D.B. were involved in the diagnostic workup, including evaluation for underlying monoclonal gammopathy and multidisciplinary management. S.P. coordinated the case and takes responsibility for the overall integrity of the work. All authors reviewed and approved the final version of the manuscript.

## Data Availability

Original data generated and analyzed for this case report are included in this published article.

## References

[1] Hirata Y, Ishizu H, Ouchi N. Insulin autoimmunity in a case of spontaneous hypoglycemia. J Jpn Diabetes Soc. 1970;13:312‐320.

[2] Uchigata Y, Hirata Y, Iwamoto Y. Drug-induced insulin autoimmune syndrome. Diabetes Res Clin Pract. 2009;83(1):e19‐e20. 19070385 10.1016/j.diabres.2008.10.015

[3] Lupsa BC, Chong AY, Cochran EK, Soos MA, Semple RK, Gorden P. Autoimmune forms of hypoglycemia. Medicine (Baltimore). 2009;88(3):141‐153. 19440117 10.1097/MD.0b013e3181a5b42e

[4] Censi S, Mian C, Betterle C. Insulin autoimmune syndrome: from diagnosis to clinical management. Ann Transl Med. 2018;6(17):335. 30306074 10.21037/atm.2018.07.32 PMC6174196

[5] Ismail AAA . The insulin autoimmune syndrome (IAS) as a cause of hypoglycaemia: an update on the pathophysiology, biochemical investigations and diagnosis. Clin Chem Lab Med. 2016;54(11):1715‐1724. 27071154 10.1515/cclm-2015-1255

[6] Church D, Cardoso L, Kay RG, et al Assessment and management of anti-insulin autoantibodies in varying presentations of insulin autoimmune syndrome. J Clin Endocrinol Metab. 2018;103(10):3845‐3855. 30085133 10.1210/jc.2018-00972 PMC6179165

[7] Cappellani D, Macchia E, Falorni A, Marchetti P. Insulin autoimmune syndrome (Hirata disease): a comprehensive review fifty years after its first description. Diabetes Metab Syndr Obes. 2020;13:963‐978. 32308449 10.2147/DMSO.S219438 PMC7136665

[8] Rajkumar SV, Dimopoulos MA, Palumbo A, et al International myeloma working group updated criteria for the diagnosis of multiple myeloma. Lancet Oncol. 2014;15(12):e538‐e548. 25439696 10.1016/S1470-2045(14)70442-5

[9] Redmon JB, Nuttall FQ. Autoimmune hypoglycemia. Endocrinol Metab Clin North Am. 1999;28(3):603‐vii. 10500933 10.1016/s0889-8529(05)70090-6

[10] Lichtman MA, Balderman SR. Unusual manifestations of essential monoclonal gammopathy. II. Simulation of the insulin autoimmune syndrome. Rambam Maimonides Med J. 2015;6(3):e0027. 26241232 10.5041/RMMJ.10212 PMC4524400

